# An open-label pilot study on preventing glucocorticoid-induced diabetes mellitus with linagliptin

**DOI:** 10.1186/s13256-018-1817-6

**Published:** 2018-10-04

**Authors:** Yoshia Miyawaki, Ken-Ei Sada, Yosuke Asano, Keigo Hayashi, Yuriko Yamamura, Sumie Hiramatsu, Keiji Ohashi, Michiko Morishita, Haruki Watanabe, Yoshinori Matsumoto, Katsue Sunahori-Watanabe, Tomoko Kawabata, Jun Wada

**Affiliations:** Department of Nephrology, Rheumatology, Endocrinology and Metabolism, Okayama University Graduate School of Medicine, Dentistry and Pharmaceutical Sciences, 2-5-1 Shikata-cho, Kitaku, Okayama City, 700-8558 Japan

**Keywords:** Diabetes, Glucocorticoid, Dipeptidyl peptidase-4 inhibitor

## Abstract

**Background:**

Numerous patients develop diabetes in response to glucocorticoid therapy. This study explored the efficacy, safety, and preventive potential of the dipeptidyl peptidase-4 inhibitor, linagliptin (TRADJENTA®), in the development of glucocorticoid-induced diabetes mellitus.

**Methods:**

From December 2014 to November 2015, we recruited non-diabetic Japanese patients scheduled for treatment with daily prednisolone ≥20 mg. Enrolled patients had at least one of following risk factors for glucocorticoid-induced diabetes mellitus: estimated glomerular filtration rate ≤ 60 mL/minute/1.73 m^2^; age ≥ 65 years; hemoglobin A1c > 6.0%. A daily dose of 5 mg of linagliptin was administered simultaneously with glucocorticoid therapy. The primary outcome was the development of glucocorticoid-induced diabetes mellitus. Additional orally administered hypoglycemic medications and/or insulin injection therapy was initiated according to the blood glucose level.

**Results:**

Four of five patients developed glucocorticoid-induced diabetes mellitus within 1 week of glucocorticoid treatment. For 12 weeks, two of the four patients with glucocorticoid-induced diabetes mellitus required orally administered medications, but no patients required insulin. Blood glucose levels before breakfast and lunch tended to decrease with time; the median glucose levels before breakfast were 93 and 79.5 mg/dL at 1 and 3 weeks, respectively. Two patients experienced mild hypoglycemia around 2 weeks. Glucose levels after lunch remained high throughout all 4 weeks despite decreasing the glucocorticoid dosage.

**Conclusions:**

Linagliptin may be insufficient to prevent the development of glucocorticoid-induced diabetes mellitus but has the potential to reduce the requirement for insulin injection therapy. Treatment of glucocorticoid-induced diabetes mellitus was continued for at least 1 month and fasting hypoglycemia in early morning should be monitored after 2 weeks.

**Trial registration:**

This trial was registered 02 November 2014 with UMIN Clinical Trials Registry (no. 000015588).

## Background

Glucocorticoids (GCs) remain a necessary component of therapy for many diseases. However, from 2 to 30% of patients treated with these drugs develop GC-induced diabetes mellitus (GC-DM) [1, 2]. A previous report showed that an older age, higher hemoglobin A1c (HbA1c) level, and lower estimated glomerular filtration rate (eGFR) were independent risk factors for the development of GC-DM, and 78% of patients with any of these risk factors developed GC-DM [3].

GC-induced hyperglycemia is caused by the development of insulin resistance and beta cell dysfunction [4–6]. Elevation of postprandial blood glucose levels is a major characteristic of GC-DM [7] and insulin is recommended as the drug of choice for the treatment of GC-induced hyperglycemia [8]. A previous study reported that insulin injection therapy was required in 50% of renal transplant recipients treated with high-dose GCs [9]. However, insulin injection therapy degrades the quality of life in patients with diabetes. Thus, treatment options without insulin injection therapy are desired in patients with GC-DM. A recent study indicated that nateglinide and acarbose were treatment options for GC-DM and improved postprandial hyperglycemia [10].

Incretin-related drugs, such as dipeptidyl peptidase-4 (DPP-4) inhibitors and glucagon-like peptide-1 (GLP-1) receptor agonists, lower blood glucose in patients with type 2 diabetes through a transient glucose-dependent stimulation of insulin and suppression of glucagon secretion [11]. A previous report showed that a GLP-1 receptor agonist prevented GC-induced glucose intolerance and islet cell dysfunction in healthy humans [12]. DPP-4 inhibitors may have similar effects but have not yet been assessed clinically. GCs cause insulin resistance via glucose transporter 4 (GLUT4) [13, 14] and endogenous glucose production directly by leading to an increment in gluconeogenesis [15]. Because DPP-4 inhibitors upregulate GLUT4 translocation [16, 17] and lower blood glucose through a glucose-dependent manner, it could be a treatment option for GC-DM. In this pilot study, we explored the DPP-4 inhibitor, linagliptin, for its efficacy and safety in preventing the development of GC-DM in non-diabetic patients. We also assessed the clinical course of GC-DM in non-diabetic patients.

## Methods

### Patients

From December 2014 to November 2015, we recruited consecutive Japanese in-patients in the Nephrology, Endocrinology, Metabolism, or Rheumatology units of Okayama University Hospital without diabetes mellitus (DM) who were scheduled for treatment with daily prednisolone doses of ≥20 mg. The definition of DM was a fasting blood glucose level over 126 mg/dL, postprandial glucose level over 200 mg/dL twice, or a history of orally administered hypoglycemic agent use. Enrolled patients had at least one of the following risk factors for GC-DM: (1) eGFR ≤ 60 mL/minute/1.73m^2^; (2) age ≥ 65 years; or (3) HbA1c (National Glycohemoglobin Standardization Program equivalent value) > 6.0% [3]. Patients were excluded for age < 20 years, an inability of internal use of the DPP-4 inhibitor, pregnancy, GC pulse therapy was scheduled, previous use of orally administered GCs, and malignancy.

### Treatment protocol

Eligible patients received a daily dose of 5 mg of linagliptin for up to 12 weeks from the initiation of GC therapy. Thereafter, patients continued taking the DPP-4 inhibitor if the attending physician determined that it was necessary. All patients also ingested a calorie-restricted diet: daily calorie intake, 30 kcal per ideal body weight (kg). Patients were permitted to receive another orally administered hypoglycemic agent, such as alpha-glucosidase inhibitors (α-GI) and/or glinides, if their postprandial blood glucose level exceeded 200 mg/dL after the diagnosis of GC-DM. One week after the addition of an α-GI and/or glinide, patients received insulin injection therapy if their postprandial blood glucose level remained over 200 mg/dL. Whenever the participants’ postprandial blood glucose level exceeded 300 mg/dL, insulin injection therapy was begun.

### Data collection

Enrolled patients were reviewed for a family history of diabetes, underlying disease, comorbidities such as hypertension and dyslipidemia, and laboratory data. Following the initiation of GC therapy, their blood glucose level was measured using a glucose meter (Medisafe®; Terumo, Japan). Preprandial (30 minutes prior to a meal) and postprandial (2 hours after a meal) blood glucose levels were measured alternately each day during admission. Patients were also evaluated at weeks 4, 8, and 12. The following data were collected and analyzed: laboratory data, treatments, and adverse effects.

### Outcome measures

The primary outcome was the development of GC-DM within 4 weeks. The definition of GC-DM was a fasting blood glucose level over 126 mg/dL, postprandial glucose level over 200 mg/dL twice, or the initiation of orally administered hypoglycemic agent use after the GC therapy. We also assessed the number of patients with concomitant use of other orally administered antidiabetic drugs and/or insulin injection therapy. Treatment-related adverse effects were recorded over the 12-week observation period and graded according to the Common Terminology Criteria for Adverse Events, version 4.0 (http://evs.nci.nih.gov/ftp1/CTCAE/About.html).

### Statistical analyses

All data are expressed as the median and range, unless otherwise specified. Statistical analyses were performed using the JMP 11 software package (SAS Institute, Cary, NC, USA).

## Results

### Patient background characteristics

**Fig. 1:**
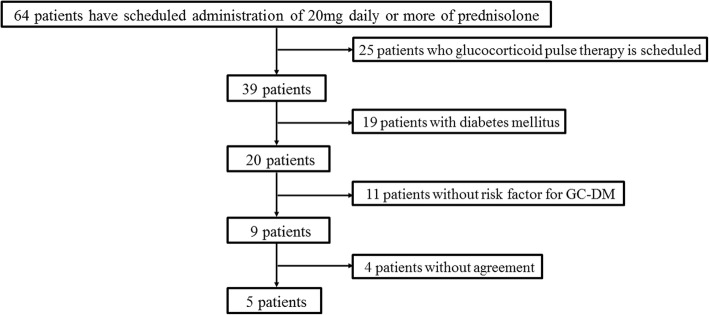
Flowchart of the study protocol. *GC-DM* glucocorticoid-induced diabetes mellitus

Of 64 candidate patients, five (three females and two males) fulfilled the inclusion criteria and were enrolled in this study. The entry procedure flowchart of the patient selection process is summarized in (Fig. 1). The characteristics of the five enrolled patients are summarized in Table 1. Median age was 72 (70–82) years. Underlying diseases included rheumatic diseases in four patients (giant cell arteritis with polymyalgia rheumatica, polymyalgia rheumatica, granulomatosis with polyangiitis, and microscopic polyangiitis) and renal disease in one patient (membranoproliferative glomerulonephritis with cryoglobulinemia). The C-reactive protein (CRP) level was elevated in all four patients with rheumatic disease, but was within the normal range in the one patient with renal disease. The median serum creatinine level was 0.7 (0.5–2.3) mg/dL and two patients exhibited eGFR of ≤60 mL/minute/1.73 m^2^. HbA1c was 5.8% (4.6–6.2) and serum glycated albumin was 14.4% (11.8–15.1). The median daily dose of prednisolone was 30 (20–50) mg.

**Table 1:** Baseline characteristics of enrolled patients

Patient no.	Gender / age	Family history of diabetes	Underlying disease	BMI	Maximum dose of PSL	CRP	eGFR	HbA1c	Triglyceride	LDL / HDL ratio
				kg/m^2^	mg/day	mg/dL	mL/min/ 1.73m^2^	%	mg/dL	
1	F / 82	Absent	Giant cell arteritis with polymyalgia rheumatica	16.3	30	4.2	47.1	5.8	94	2.2
2	F / 70	Present	Polymyalgia rheumatica	20.3	20	11.9	86.6	5.7	111	2.0
3	M / 72	Absent	Granulomatosis with polyangiitis	19.1	50	10.3	80.2	6.2	100	2.6
4	F / 77	Absent	Microscopic polyangiitis	20.5	35	11.3	16.3	5.9	118	1.1
5	M / 70	Absent	Membranoproliferative glomerulonephritis with cryoglobulinemia	18.4	30	0.16	100.2	4.6	82	0.2

*BMI* body mass index, *CRP* C-reactive protein, *eGFR* estimated glomerular filtration rate, *F* female, *HbA1c* hemoglobin A1c, *HDL* high-density lipoprotein cholesterol, *LDL* low-density lipoprotein cholesterol, *M* male, *PSL* prednisolone

### Clinical course

The one patient with renal disease did not develop GC-DM but withdrew at day 19 because of a hospital transfer. The other four patients with rheumatic disease developed GC-DM within 1 week. All patients were diagnosed as having GC-DM by the postprandial but not the fasting glucose level. All patients continued taking linagliptin for the observational period. Two of the four patients with GC-DM required additional orally administered medications (one patient received 0.5 mg of repaglinide daily and the other received 0.3 mg of voglibose daily). No patients required insulin injection therapy. The median HbA1c levels were (*N* = 3 for all) 5.4% (5.3–6.1) at 4 weeks, 5.5% (4.9–5.6) at 8 weeks, and 5.7% (5.1–5.9) at 12 weeks.

**Fig. 2:**
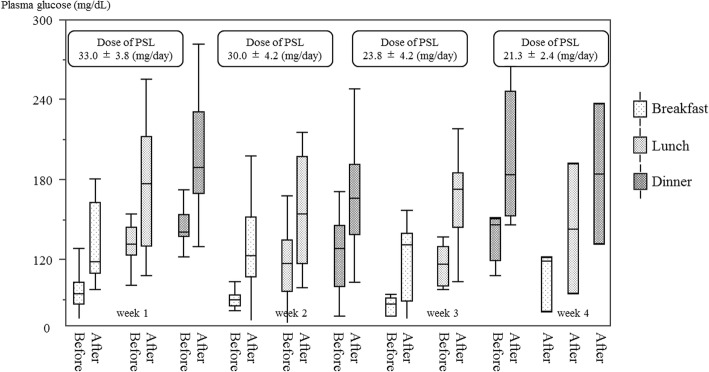
Blood glucose levels of patients before and after meals. Data are expressed as the average blood glucose level and a range before and after each meal from weeks one to four. Prednisolone doses are given as the median and range. *PSL* prednisolone

The weekly trends of glucose levels before and after each meal are shown in (Fig. 2). The glucose level before breakfast was the lowest of the day at any week. Glucose levels before breakfast and lunch tended to decrease week by week, but the glucose levels after lunch remained high throughout the study period despite decreasing the prednisolone dosage (Fig. 2).

### Safety

During the 12-week follow-up period, neither elevated serum amylase nor overt pancreatitis occurred in all cases. Grade 1 hypoglycemia occurred in two patients (one patient with GC-DM and the other without GC-DM). Hypoglycemia occurred in the morning after overnight fasting at days 13 and 14 after the initiation of GC therapy in two different patients. No other adverse effects related to the treatment occurred.

## Discussion

The principal findings of the present study are that four of five non-diabetic patients developed GC-DM in spite of the concomitant use of a DPP-4 inhibitor. Two patients required additional orally administered medications, but no patients required insulin injection therapy.

All four patients with rheumatic disease developed GC-DM within a few days. The one patient with renal disease did not develop DM up to day 19 when they withdrew from the study. The proportion of patients that developed GC-DM in the present population was similar to our previous observational study in spite of the concomitant use of a DPP-4 inhibitor [3]. Previous reports showed that a GLP-1 receptor agonist prevented GC-induced glucose intolerance, but that a DPP-4 inhibitor failed to improve this effect [12, 18]. These results suggest that DPP-4 inhibitors might have insufficient efficacy to prevent the development of GC-DM.

All patients that developed GC-DM exhibited increased levels of CRP at baseline. The inflammatory state is known to exacerbate insulin resistance [19]. Therefore, it may be more difficult to prevent the development of GC-DM in patients with rheumatic disease compared to those without inflammation. Because elevated HbA1c levels are a risk factor for GC-DM, a low titer of HbA1c at baseline may be related to the onset of GC-DM.

DPP-4 inhibitors may decrease the requirement for insulin injection therapy for the treatment of GC-DM. Although two patients required orally administered medications such as a glinide or an α-GI, no patients required insulin injection therapy in the present study. A study from 33 years ago reported that insulin injection therapy was required in 50% of renal transplant recipients who were given high-dose GC therapy [9] while a recent study indicated that nateglinide and acarbose improved postprandial hyperglycemia in patients with GC-DM [10]. These findings suggested that concomitant use of a glinide, α-GI, and/or a DPP-4 inhibitor enable tight control of postprandial hyperglycemia in patients with GC-DM without the need for insulin injection therapy.

All patients with GC-DM were diagnosed by postprandial glucose levels within a few days. Overt hyperglycemia developed between 21 and 270 days after transplantation (mean 65 days) [20] but measurement points were not described in that study. Postprandial glucose levels increased from the first day of GC administration in this study. Thus, GC-DM can be diagnosed within a few days if postprandial glucose levels are measured daily after the initiation of GC therapy. Postprandial hyperglycemia from lunch to dinner remained high after 4 weeks. These results indicated that treatment of GC-DM was necessary for at least 1 month once GC-DM developed.

In contrast to hyperglycemia, fasting blood glucose levels decreased week by week, and two patients experienced mild hypoglycemia after approximately 2 weeks. Only one of these patients had GC-DM. A previous report showed that glucose levels before breakfast were lowest among patients using continuous glucose monitoring 4 weeks after initiation of GC therapy [21]. Another recent report showed that fasting blood glucose levels were significantly lower in patients with rheumatic disease treated with GC than those without GC, who had not been diagnosed with DM previously [22]. In the present study, increase of insulin secretion by DPP-4 inhibitors via night hyperglycemia might cause fasting hypoglycemia in the morning. These results suggested that fasting hypoglycemia in the early morning after 2 weeks of GC therapy was a notable adverse effect in patients with GC-DM with DPP-4 inhibitors.

We acknowledge that our study is subject to several limitations. Because this was a pilot study, the sample size was small and data collection was confined to 3 months. Thus, the results were insufficient to confirm the efficacy and safety of linagliptin for GC-DM. However, we concluded that the extension of the study was not ethical because four of the five patients developed GC-DM. It is possible that the need for insulin injection therapy can be reduced or eliminated. Furthermore, it appears that fasting hypoglycemia in the early morning after 2 weeks of GC therapy should be monitored. Furthermore, we did not screen enrolled patients with the 75-g oral glucose tolerance test (OGTT) in this study. Actually, OGTT is not always performed in the clinical setting of patients who need GC treatment without clinically apparent DM, even if it is better to screen by OGTT. We have already evaluated that an older age, higher HbA1c level, and lower eGFR were independent risk factors for the development of GC-DM in such a situation [3]. We confirmed that GC-DM really developed to a high rate and DPP-4 inhibitors might have insufficient efficacy to prevent the development of GC-DM in this study.

## Conclusions

The results of this preliminary study indicated that linagliptin may be insufficient to prevent the development of GC-DM, but may decrease the requirement for insulin injection therapy. Treatment of GC-DM was necessary for at least 1 month after GC-DM developed, and fasting hypoglycemia in early morning should be monitored after 2 weeks of GC therapy with DPP-4 inhibitors.
